# Traumatic Anserine Folliculosis: A Case Report

**DOI:** 10.7759/cureus.99393

**Published:** 2025-12-16

**Authors:** Dinah F AlAhmadi, Fadi Alghamdi, Reema S AlZaidi, Samar S Alwafi

**Affiliations:** 1 Medicine, King Saud Bin Abdulaziz University for Health Sciences College of Medicine, Jeddah, SAU; 2 Dermatology, King Fahad Armed Forces Hospital, Jeddah, SAU

**Keywords:** case report, follicular papules, frictional dermatosis, topical retinoids, traumatic anserine folliculosis

## Abstract

Traumatic anserine folliculosis (TAF) is a rare, under-recognized dermatological condition caused by repeated friction or pressure. This leads to grouped follicular papules with a rough, sandpaper-like texture, most commonly on the face or neck. It primarily affects adolescents and young adults, with pathogenesis linked to mechanical irritation causing follicular hyperkeratosis and keratin plug formation. We report the case of a young male who developed hyperpigmented follicular papules on his left cheek with a history of resting his cheek on his palm. Clinical evaluation confirmed TAF, and treatment with topical retinoids and keratolytics, along with avoidance of friction, led to marked improvement after nine months. The diagnosis of TAF is clinical and relies on recognizing the frictional history and characteristic lesions, distinguishing it from conditions like keratosis pilaris (KP) and lichen spinulosus. Management centers on educating patients, eliminating trauma, and using topical retinoids or keratolytics, with generally excellent outcomes when identified and treated early.

## Introduction

Traumatic anserine folliculosis (TAF) is a rare yet under-recognized dermatological condition characterized by multiple, closely grouped follicular papules predominantly affecting areas such as the chin, jawline, and neck [1]. These papules often present with a sandpaper-like texture upon palpation. TAF primarily affects children and adolescents, with friction and pressure identified as key etiological factors [2]. Despite its benign nature, TAF is frequently misdiagnosed as other follicular disorders such as comedonal acne, keratosis pilaris (KP), or lichen spinulosus due to overlapping clinical features [3].

Although TAF is more common than recognized, it remains underreported, with only a limited number of cases described in the literature [3,4].

In this case report, we present a 21-year-old male with itchy lesions localized to his left cheek. We highlight the clinical presentation, diagnostic challenges, and management options of TAF. The objective of this case report is to emphasize the importance of recognizing this condition and differentiating it from other similar follicular disorders to avoid unnecessary investigations, ensure effective treatment, and improve patient education.

## Case presentation

A 21-year-old male presented with an intermittently itchy lesion on his left cheek. The lesion had been present for five years. He acknowledged resting in a particular position (resting his palm on his left cheek), which led to prolonged localized pressure and friction in the area while watching television. There was no history of atopy or family history of a similar condition.

**Figure 1:**
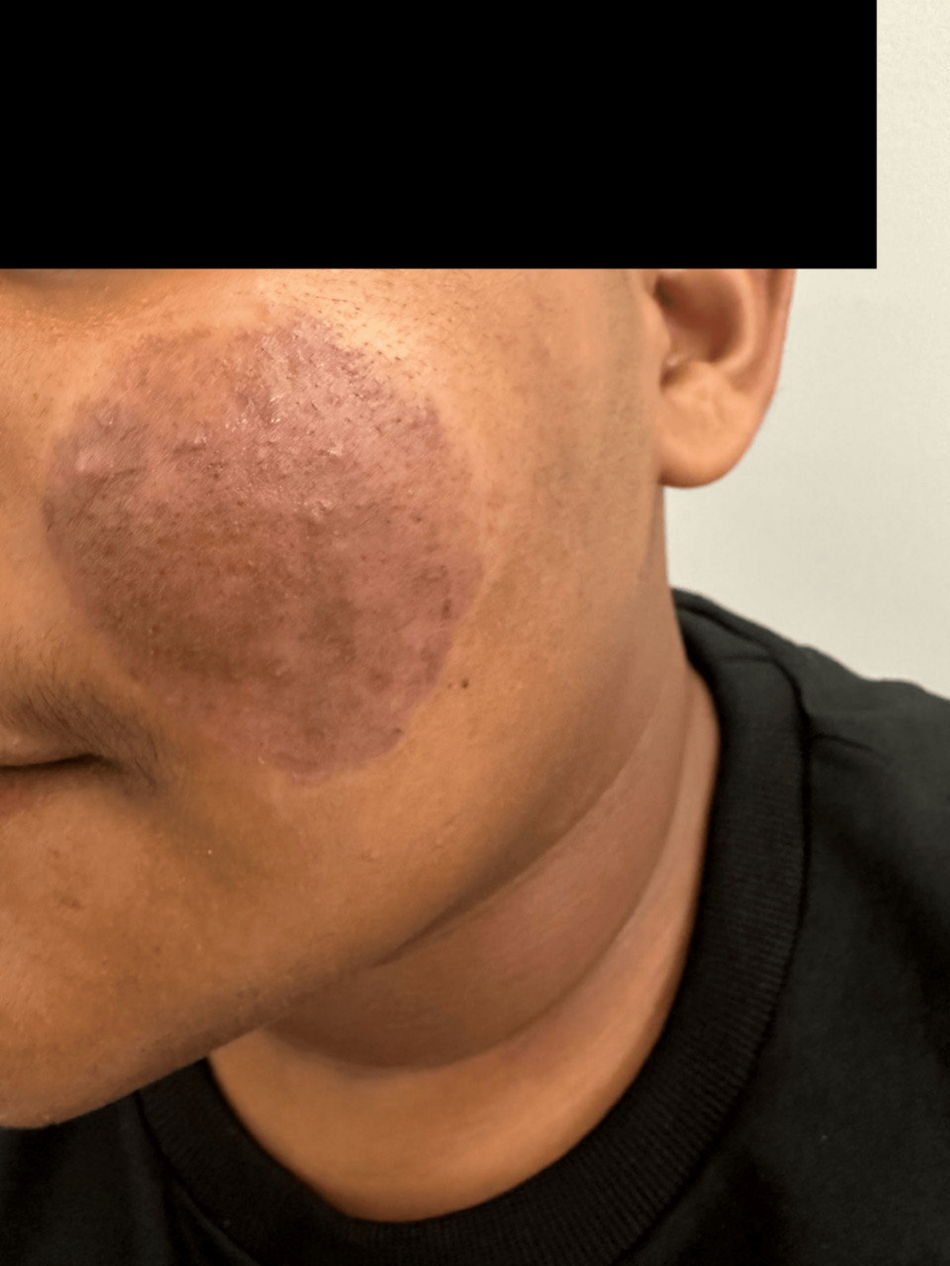
Multiple hyperpigmented follicular papules overlying a well-demarcated hyperpigmented plaque on the cheek.

Cutaneous examination revealed multiple hyperpigmented follicular papules overlying a well-demarcated hyperpigmented plaque (Figure 1). The oral mucosa, hair, and nails were not involved. The systemic review of systems was unremarkable. Differential diagnoses of KP, lichen spinulosus, trichostasis spinulosa, trichodysplasia spinulosa, disseminated and recurrent infundibular folliculitis, and comedonal acne were considered.

**Figure 2:**
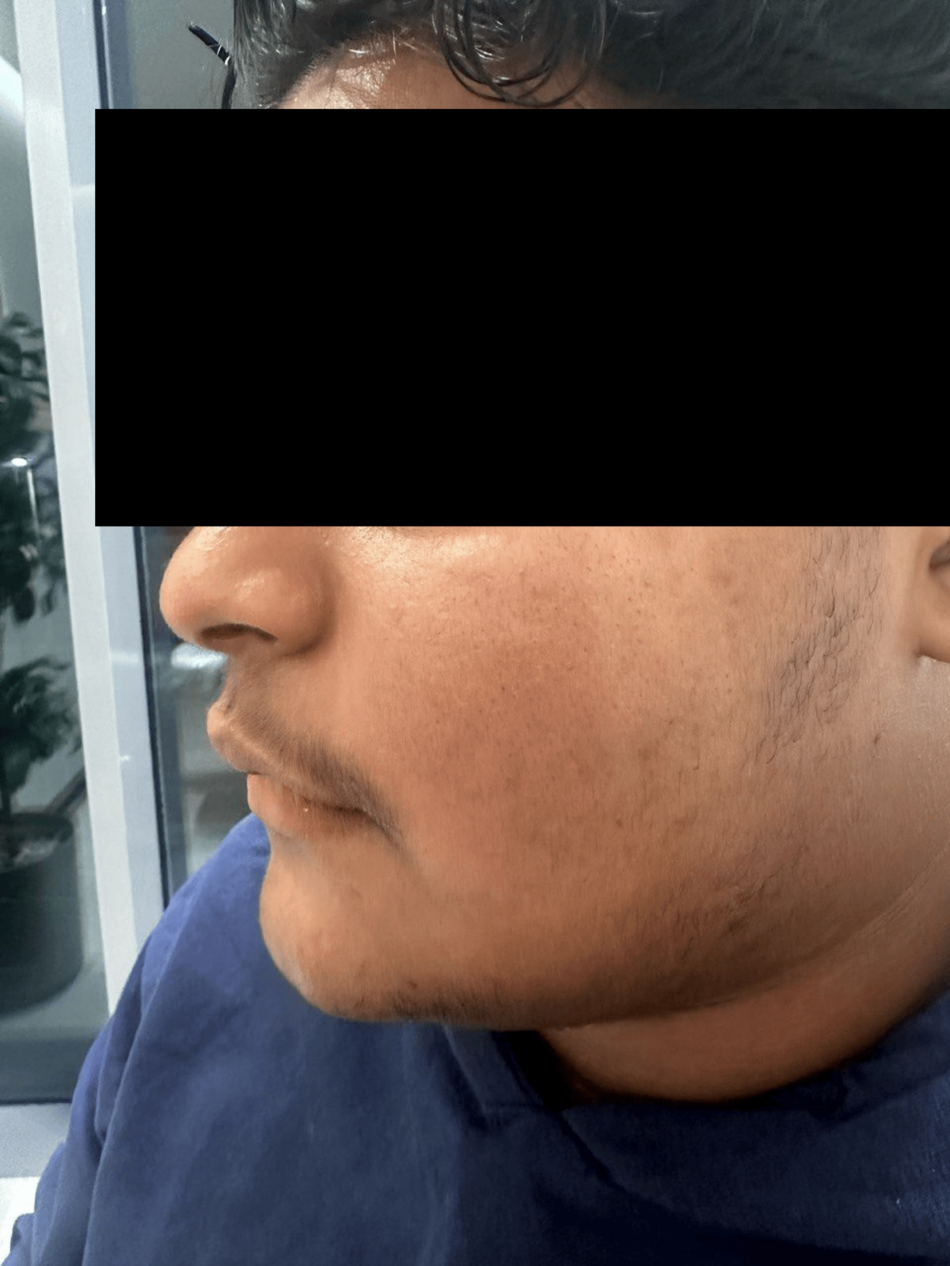
Improvement after treatment with topical keratolytics.

The patient was advised to avoid friction or trauma to the affected area and was treated with topical tretinoin 0.05% cream combined with 4% hydroquinone cream. He showed gradual improvement after nine months of treatment with topical keratolytics along with the removal of etiological factors (Figure 2).

Given the characteristic history of mechanical irritation, the typical follicular morphology, and the improvement after eliminating the pressure trigger and applying topical retinoid therapy, a skin biopsy was deemed unnecessary to avoid an invasive procedure. Accordingly, a diagnosis of TAF was established.

## Discussion

TAF is a rare dermatological condition characterized by firm, sandpaper-like follicular papules that typically occur in areas prone to friction and pressure, such as the chin, cheeks, and jawline. It predominantly affects adolescents and young adults [4]. First described by Padilha-Gonçalves A in 1979, TAF is often associated with habitual behaviors such as resting the face on the hands or wearing tight headgear [5]. Diagnosis is primarily clinical, supported by dermoscopic findings of dilated follicles with keratin plugs, perifollicular erythema, and white scales. Histopathological examination reveals dilated follicular infundibula filled with keratin and minimal inflammatory infiltrate, distinguishing it from other follicular disorders. Management includes patient education to minimize friction, the use of topical keratolytics such as salicylic acid or urea, and, in persistent cases, mild topical corticosteroids to reduce inflammation [6].

The differential diagnosis of TAF includes several follicular disorders that share overlapping clinical features. These include KP, lichen spinulosus, trichostasis spinulosa, trichodysplasia spinulosa, disseminated and recurrent infundibular folliculitis, and comedonal acne.

KP typically presents as small, rough, follicular papules with a sandpaper-like texture, often affecting the extensor surfaces of the upper arms, thighs, and buttocks. KP is associated with keratin plug formation and may be exacerbated by dry skin [7]. Treatment includes the use of keratolytic agents such as salicylic acid, urea, and topical retinoids to reduce follicular plugging [8].

Lichen spinulosus is characterized by grouped, keratotic, symmetric papules that resemble goosebumps, commonly found on the trunk and extremities. Unlike KP, lesions in lichen spinulosus may resolve spontaneously [7]. Treatment options include emollients, keratolytics, and topical corticosteroids to alleviate pruritus and reduce inflammation [9].

Trichostasis spinulosa presents as multiple follicular papules resembling comedones with tufts of fine vellus hair projections, typically on the face or back [7]. Extraction and topical retinoids are commonly employed to manage trichostasis spinulosa [10].

Trichodysplasia spinulosa is a rare condition associated with immunosuppression and polyomavirus infection. It presents with spiny papules predominantly on the face and is associated with trichodysplasia, characterized by abnormal hair follicle development [6]. Management involves antiviral therapy and reduction of immunosuppressive medications [11].

Disseminated and recurrent infundibular folliculitis is characterized by widespread, pruritic, follicular papules and pustules, primarily affecting the trunk and extremities. It is often chronic and recurrent, requiring long-term management with oral and topical antibiotics, retinoids, or corticosteroids [12].

Comedonal acne presents as flesh-colored to whitish papules of 1-3 mm in diameter, commonly involving the face and trunk. Management includes anti-acne therapy [6].

Differentiating TAF from these conditions is essential to avoid misdiagnosis and unnecessary treatment. The patient’s history of localized pressure and friction, clinical examination findings, and, when needed, histopathological evaluation can aid in accurate diagnosis and appropriate management.

## Conclusions

TAF is characterized by grouped follicular papules overlying a hyperpigmented plaque on the face and neck, most commonly caused by frictional trauma. TAF is clinically diagnosed and can be differentiated from other similar conditions by the history of friction and the characteristic lesions. Management involves treatment with topical retinoids and the avoidance of friction or trauma. Clinician awareness of TAF is essential for accurate diagnosis and management, helping to avoid unnecessary invasive procedures and enhance patient care.
